# Education, Altitude, and Humidity Can Interactively Explain Spatial Discrepancy and Predict Short Stature in 213,795 Chinese School Children

**DOI:** 10.3389/fped.2019.00425

**Published:** 2019-10-30

**Authors:** Jia Ma, Zhixin Zhang, Wenquan Niu, Jie Chen, Sihui Guo, Shufang Liu, Yanhui Dong, Zhaogeng Yang, Wenlai Wang, Ci Song, Jun Ma, Tao Pei

**Affiliations:** ^1^Graduate School, Beijing University of Chinese Medicine, Beijing, China; ^2^Department of Pediatrics, China-Japan Friendship Hospital, Beijing, China; ^3^Institute of Clinical Medical Sciences, China-Japan Friendship Hospital, Beijing, China; ^4^State Key Laboratory of Resources and Environmental Information System, Institute of Geographic Sciences and Natural Resources Research, Chinese Academy of Sciences, Beijing, China; ^5^Institute of Child and Adolescent Health and School of Public Health, Peking University, Beijing, China

**Keywords:** short stature, prevalence, risk factor, spatial discrepancy, prediction

## Abstract

**Backgrounds and Objectives:** The north–south height distinctions in Chinese children suggest that some geographical–climatic factors could determine height variation of short stature. In a national health survey, we aimed to explore the spatial distribution of short stature on city scales, and detect its socio-economic and geographical–climatic factors.

**Methods:** Data on the prevalence of short stature were obtained from a 2014 cross-sectional survey of China (CNSSCH). In total, 213,795 Han Chinese students aged 7–18 years, from 106 cities across 30 provinces, were included. Both China and World Health Organization (WHO) growth references were adopted to define short stature.

**Results:** A spatial clustering was apparent in the distribution of short stature. After multivariable adjustment, altitude and humidity significantly increased the risk of high prevalence in short stature, according to the WHO (odds ratio [OR] = 1.61 and 1.26, 95% confidence interval [CI]: 1.20–2.17 and 1.03–1.54) and China (OR = 1.54 and 1.26; 95% CI: 1.15–2.05 and 1.02–1.55) growth references. Additionally, education significantly decreased the risk of high prevalence in short stature according to the WHO (OR = 0.40; 95% CI: 0.22–0.74) and China (OR = 0.42; 95% CI: 0.22–0.77) growth references. Combining both altitude >400 m and education <9 years, as well as education <9 years and humidity >70%, received the largest effect-size estimate, and significance retained after multivariable adjustment.

**Conclusions:** Our findings indicate that high altitude and humidity increased the risk of high prevalence in short stature, high education was associated with low prevalence. Additionally, we observed possible interactions between education and altitude/humidity. They may interactively explain spatial discrepancy and predict short stature in Chinese school children. Further validations are necessary.

## Introduction

Organisms constantly adapt to external environment, for example, as people shift from a hunting–gathering lifestyle into a society based on agriculture and animal husbandry, adaptation changes such as height, fatty acid desaturase enzymes and hemoglobin concentration, begin taking place (1). For example, Peruvians' adaptation to a plateau environment with oxygen deficit and ultraviolet exposure are manifested in their short stature and thick skin (2). In addition, some genomic differences have been identified in omega-3 polyunsaturated fatty acid-related regions in Inuit who have adapted to a seafood diet (3). Furthermore, some lowlanders who moved to plateau region had elevated hemoglobin concentration upon, this phenomenon has been confirmed by many studies, as a mechanism of adaptation to oxygen starvation (4–6).

There is a spatial distribution discrepancy between stunting and short stature worldwide, with Central and Western Africa, as well as South Asia being particularly severe places (33.7 and 35%, respectively) (7). A study has shown that due to limited food, high temperature and humidity, Western Africa tropical rainforest residents tended to have short stature and reached puberty earlier. In this situation, short stature was not a pathological state, but a protective mechanism for environmental acclimatization (8). In our previous study (9), we also identified a specific spatial distribution of short stature in Mainland China. We try to explain why? What factors account for this spatial distribution of short stature in mainland of China? Socio-economic factors could also be crucial determinants of short stature (10, 11); however, height variations exist between diverse races and regions (12, 13). Due to the examples of environmental acclimatization that mentioned above, we assume that geographical–climatic factors also have great influence to short stature. In this paper, we will explore the spatial distribution of prevalence of short stature in smaller scale (i.e., city scale), and detect the socio-economic and geographical–climatic factors of short stature in mainland of China. We believe that the results of research can help us understand the environmental acclimatization better and it is significant contribution to prevention and control of short stature for government.

## Methods

### Datasets

Data on the prevalence of short stature came from the 2014 Chinese National Survey on Students Constitution and Health (CNSSCH)—a cross-sectional survey in China. Details of the sampling and measurements have been previously published (9, 14–16). All participants were collected by stratified cluster sampling, the characteristics of participating we have reported detailedly in the previous study (9).

Socio-economic data including sex ratio (girls = 100), proportion of non-agricultural household registration (%) and education duration (years) were collected from China's 2010 Census data (17). Geoclimatic data covered altitude (m), annual mean relative humidity (%), annual mean sunshine (hours), annual mean temperature (°C), annual mean wind speed (m/s) and annual mean rain (mm), which were obtained from the National Earth System Science Data Sharing Infrastructure (National Science and Technology Infrastructure of China)^1^ The annual mean relative humidity, annual mean sunshine, annual mean temperature, annual mean wind speed, and annual mean rain data were annual data from 2000 to 2010, i.e., the data represent an average over 11 years. Data conversion was performed during the logistic regression analysis, and the details are as follows: altitude/250, humidity/3, sunshine/200, temperature/3, rain/100, and wind/5.

In our previous study (9), we observed that there are sex and rural-urban differences in prevalence of short stature. As reported by other researchers, education, altitude, humidity, temperature, and rain were significantly associated with short stature (2, 18–23). Additionally, it is well known that sunshine can help synthesize vitamin D (the major source of vitamin D in the body), which has a close relation with short stature (24, 25). Hence, above factors are incorporated in the present analysis.

### Anthropometric Measurements

Children whose height fell below the third centile, compared to the same age, gender and ethnic population were defined as short stature (26). China and the World Health Organization (WHO) growth references were used for the diagnosis of short stature (27, 28).

We calculated the prevalence of short stature in a city-scale. Overall, 213,795 Han students between the ages of 7–18, in 106 cities of 30 provinces (except for Tibet, Hong Kong, Macao and Taiwan), were included in our study.

### Statistical Analyses

To improve the comparability between cities, we used direct age-standardized prevalence and direct age-gender-standardized prevalence according to the China and WHO growth references, respectively, in the China 2000 Census (29, 30). Moran's I, Getis-Ord Gi^*^ and Local Moran's I were assessed for the spatial disparities between different cities and the results were also displayed on maps (31–33) using the ArcMap software 10.2 (ESRI, Redlands, California, USA).

We then changed the prevalence of short stature from a continuous variable into a binary variable and the national prevalence of short stature (3.70 and 2.69% according to the China growth reference and WHO growth reference, respectively) as the cut-off value that divided 106 cities into two groups, where one was higher and the other lower than the overall prevalence. We performed a rank-sum test to compare the differential factors between the two groups (as the data did not fit a normal distribution). Logistic regression was used to detect significant risk factors before adjustment, then we chose forward, backward, stepwise regression to filter the variables. And multivariable adjustment by sex ratio, sunshine, wind speed and temperature (duo to proportion of non-agricultural household registration is related with education, rain is related with humidity, we didn't include these two factors in multivariable adjustment). STATA software special edition (version 14.0, Stata Corp, TX, USA) was used for statistical analyses.

We used significant risk factors to draw a prediction nomogram using R-language (version 3.5.2). Predictive accuracy was assessed by the concordance index (C-index) and defined as the area under the receiver operating characteristics curve. The sample size was estimated using the PS (Power and Sample Size Calculations) software Release 3.0. A *p*-value <0.05 was considered statistically significant.

## Results

### Spatial Distribution of Short Stature

**Figure 1:**
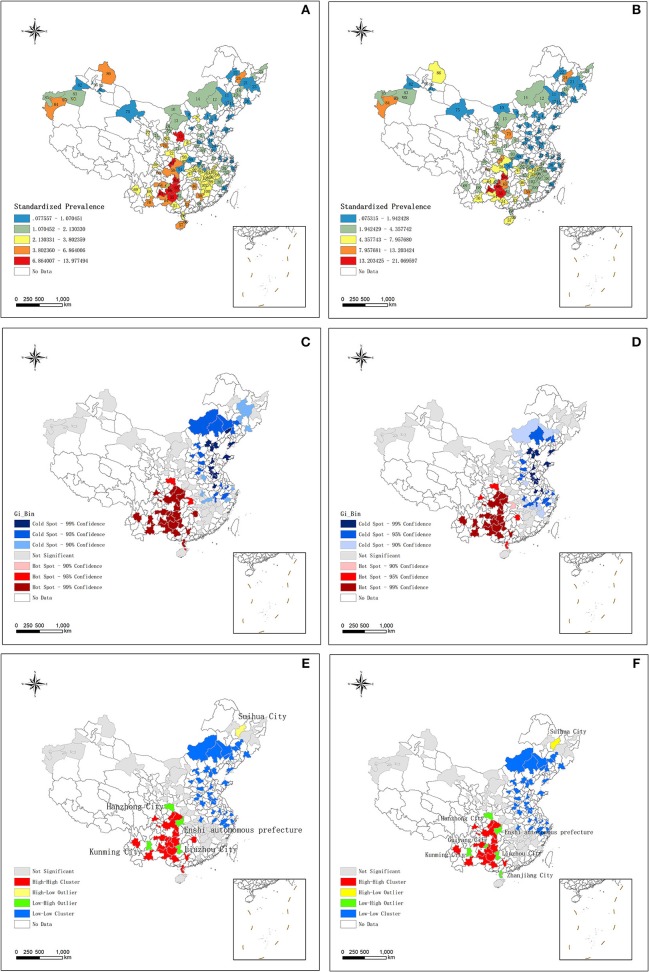
**(A)** The distribution of short stature standardized prevalence according to WHO growth reference. **(B)** The distribution of short stature standardized prevalence according to China growth reference. **(C)** The result of Hotspot Getis-Ord, Gi^*^ about short stature according to WHO growth reference. **(D)** The result of Hotspot Getis-Ord, Gi^*^ about short stature according to China growth reference. **(E)** The results of Anselin Local Moran's I about short stature according to WHO growth reference. **(F)** The results of Anselin Local Moran's I about short stature according to China growth reference. WHO, World Health Organization.

There was a spatial autocorrelation (clustering) in the distribution of short stature. The Moran's Index was 0.369 (*p* < 0.001) and 0.330 (*p* < 0.001) as assessed by the WHO and China growth references, respectively. Moran's I value close to positive one means there are existed a clustering (9, 31). The prevalence of short stature is displayed on the maps in Figures 1A,B and Supplementary Table 1. The Getis-Ord Gi^*^ results revealed a hotspot of short stature in the southwest of China, and a cold spot area in the northeast of China, indicating a similar pattern that was consistent with our previous study (Figures 1C,D and Supplementary Table 2) (9). Positive Gi ^*^ Z-scores means that there is a clustering of high prevalence of disease, scores close 0 represent no clustering and negative scores manifest clustering of low prevalence of disease (9, 33). The Anselin Local Moran I revealed a high–high cluster in southwest China, and a low–low cluster in northeast China. Notably, we discovered a high–low outlier in Suihua City and a low–high outlier in Hanzhong City, Kunming City, the Enshi Autonomous Prefecture and Liuzhou City, according to both growth references. Furthermore, there was an additive low–high outlier in Guiyang City and Zhanjiang City as assessed by the China growth reference (Figures 1E,F and Supplementary Table 3). The Anselin Local Moran I is a spatial statistical method that focuses on exploring the relationship between clusters with neighboring clusters (inter-cluster variation) (32). The high–high cluster and the low–low cluster are relatively easy to understand, they mean that there are a cluster of high prevalence or low prevalence of disease. The high–low outlier indicate the area where have the high prevalence of disease is surrounded by the pleases where have the low prevalence of disease. The low–high outlier is the opposite situation.

### Identification of Risk Factors

The characteristics of the two groups are presented in Table 1. The proportion of non-agricultural household registration, education duration, altitude, annual mean relative humidity, annual mean sunshine, annual mean temperature, annual mean wind speed and annual mean rain, except for the sex ratio, showed that the differences between the two groups were quite remarkable (all *p* < 0.05), according to both growth references. The effect-size estimates of multiple factors associated with the risk of short stature before adjustment, according to the two growth references, are shown in Table 2. Comparing the lower than overall prevalence group showed that the unadjusted risk prediction of non-agricultural household, education duration, humidity, sunshine, temperature, wind speed, rain were significantly associated with short stature according to the two growth references. Then, we used the forward, backward and stepwise methods to screen the variables. After multivariable adjustment, only humidity, education duration and altitude were significant, according to the two growth references (Table 3).

**Table 1:** The baseline characteristics of study participants using two different growth references.

**Variables**	**WHO reference**			**China reference**		
	**Lower than overall prevalence**	**Higher than overall prevalence**	***P***	**Lower than overall prevalence**	**Higher than overall prevalence**	***P***
Sex ratio (girls = 100)	104.97 (103.24, 107.26)	105.8 (103.27, 108.34)	0.330	105.01 (103.24, 107.28)	105.79 (103.04, 108.05)	0.473
Proportion of non-agricultural household registration (%)	34.17 (26.56, 45.5)	24.05 (15.82, 33.73)	<0.001	34.12 (26.30, 45.50)	23.88 (15.81, 34.16)	<0.001
Education duration (years)	9.29 (8.81, 10.09)	8.75 (7.98, 9.07)	<0.001	9.27 (8.81, 10.09)	8.71 (7.98, 9.17)	<0.001
Altitude (m)	245.65 (110.99, 1,024.29)	628.27 (204.24, 1,167.81)	0.040	238.55 (106.84, 1,024.29)	657.18 (205.74, 1,174.63)	0.018
Annual mean relative humidity (%)	64.36 (54.90, 76.2)	80.27 (75.80, 83.16)	<0.001	64.50 (54.89, 76.77)	80.47 (75.75, 83.16)	<0.001
Annual mean sunshine (hours)	2,152.05 (1,771.35, 2,560.03)	1,641.82 (1,368.44, 2,084.11)	<0.001	2,142.54 (1,771.35, 2,560.03)	1,640.15 (1,344.80, 2,086.73)	<0.001
Annual mean temperature (°C)	12.89 (7.03, 16.29)	15.79 (12.31, 17.32)	0.011	12.97 (7.03, 16.33)	15.45 (12.24, 17.24)	0.026
Annual mean wind speed (m/s)	22.02 (17.97, 26.36)	17.31 (15.02, 19.62)	<0.001	22.25 (17.97, 26.42)	17.18 (14.95, 19.60)	<0.001
Annual mean rain (mm)	709.41 (449.75, 1,541.05)	1,773.10 (1,405.86, 2,039.10)	<0.001	726.71 (449.75, 1,551.53)	1,743.37 (1,262.15, 2,028.19)	<0.001

*WHO, World Health Organization. Data are expressed as interquartile range. P-value was calculated by the rank-sum test*.

**Table 2:** The unadjusted risk prediction by logistic regression according to two growth references.

**Variables**	**WHO reference**			**China reference**		
	**OR**	**95% CI**	***P***	**OR**	**95% CI**	***P***
Sex ratio	1.02	0.92–1.11	0.747	1.01	0.91–1.11	0.882
Non-agricultural household	0.94	0.91–0.97	0.001	0.94	0.91–0.98	0.001
Education years	0.32	0.17–0.57	<0.001	0.32	0.18–0.57	<0.001
Altitude	1.11	0.97–1.28	0.129	1.13	0.98–1.30	0.090
Humidity	1.28	1.13–1.45	<0.001	1.27	1.12–1.44	<0.001
Sunshine	0.70	0.58–0.85	<0.001	0.70	0.58–0.85	<0.001
Temperature	1.38	1.07–1.78	0.013	1.31	1.02–1.68	0.032
Wind speed	0.35	0.21–0.59	<0.001	0.31	0.18–0.54	<0.001
Rain	1.12	1.06–1.20	<0.001	1.11	1.05–1.18	<0.001

*WHO, World Health Organization; OR, odds ratio; 95% CI, 95% confidence interval*.

*P-values were calculated by unadjusted logistic regression*.

*We do some conversion of data when we running logistic regression analysis, the details as follows: altitude/250, humidity/3, sunshine/200, temperature/3, rain/100, wind speed/5*.

**Table 3:** The results of forward, backward, stepwise and adjusted risk prediction by logistic regression.

**WHO reference**				**China reference**			
**Variables**	**OR**	**95% CI**	***P***	**Variables**	**OR**	**95% CI**	***P***
**FORWARD REGRESSION**							
Education	0.44	0.23–0.84	0.013	Education	0.49	0.25–0.95	0.033
Rain	1.14	1.05–1.25	0.003	Humidity	1.24	1.05–1.48	0.013
Altitude	1.24	1.02–1.51	0.033	Altitude	1.22	1.01–1.48	0.042
**BACKWARD REGRESSION**							
Humidity	1.34	1.14–1.57	<0.001	Humidity	1.24	1.05–1.48	0.013
Altitude	1.25	1.03–1.52	0.025	Education	0.49	0.25–0.95	0.033
Education	0.40	0.21–0.78	0.007	Altitude	1.22	1.01–1.48	0.042
**STEPWISE REGRESSION**							
Humidity	1.34	1.14–1.57	<0.001	Humidity	1.24	1.05–1.48	0.013
Altitude	1.25	1.03–1.52	0.025	Education	0.49	0.25–0.95	0.033
Education	0.40	0.21–0.78	0.007	Altitude	1.22	1.01–1.48	0.042
**MULTIVARIABLE ADJUSTED**							
Education	0.40	0.22–0.74	0.003	Education	0.42	0.22–0.77	0.006
Humidity	1.26	1.03–1.54	0.024	Humidity	1.26	1.02–1.55	0.029
Altitude	1.61	1.20–2.17	0.002	Altitude	1.54	1.15–2.05	0.004

*WHO, World Health Organization; OR, odds ratio; 95% CI, 95% confidence interval. P-values were calculated by logistic regression*.

*We do some conversion of data when we running logistic regression analysis, the details as follows: altitude/250, humidity/3, sunshine/200, temperature/3, rain/100, wind speed/5*.

*In multivariable adjusted model, risk prediction of each adjusted factor was calculated by adjusting for the other factors (sex, sunshine, wind speed, temperature)*.

On account of the statistical significance of education, altitude and humidity affecting the prevalence of short stature, we explored the interaction of each variate under the two growth references (Table 4). As places with high altitude and high humidity do not exist, there is a negative correlation between altitude and humidity in geography, so we did not conduct any interaction between these two variates.

**Table 4:** The interaction between humidity, education and altitude in predicting short stature.

**Variables**	**WHO reference**			**China reference**		
	**OR**	**95% CI**	***P***	**OR**	**95% CI**	***P***
**UNADJUSTED**						
**Interaction between altitude and education**						
Altitude ≤ 400 m/Education>9 years	Reference			Reference		
Altitude ≤ 400 m/Education ≤ 9 years	4.00	1.16–13.82	0.028	3.38	0.97–1.78	0.057
Altitude>400 m/Education>9 years	3.43	0.95–12.39	0.060	3.43	0.95–12.39	0.060
Altitude>400 m/Education ≤ 9 years	8.40	2.41–29.23	0.001	8.40	2.41–29.23	0.001
**Interaction between education and humidity**						
Education>9 years/Humidity ≤ 70%	Reference			Reference		
Education ≤ 9 years/Humidity ≤ 70%	1.56	0.34–7.15	0.565	1.56	0.34–7.15	0.565
Education>9 years/Humidity>70%	2.96	0.79–11.09	0.107	2.96	0.79–11.09	0.107
Education ≤ 9 years/Humidity>70%	13.89	3.72–51.81	<0.001	11.88	3.22–43.75	<0.001
**MULTIVARIABLE ADJUSTED**						
**Interaction between altitude and education**						
Altitude ≤ 400 m/Education>9 years	Reference			Reference		
Altitude ≤ 400 m/Education ≤ 9 years	2.94	0.78–11.14	0.112	2.32	0.61–8.84	0.217
Altitude>400 m/Education>9 years	9.34	1.49–58.49	0.017	6.45	1.06–39.42	0.043
Altitude>400 m/Education ≤ 9 years	13.35	2.50–71.30	0.002	9.64	1.86–50.05	0.007
**Interaction between education and humidity**						
Education>9 years/Humidity ≤ 70%	Reference			Reference		
Education ≤ 9 years/Humidity ≤ 70%	1.37	0.28–6.71	0.699	1.33	0.27–6.64	0.729
Education>9 years/Humidity>70%	2.13	0.39–11.64	0.385	2.19	0.39–12.31	0.372
Education ≤ 9 years/Humidity>70%	7.18	1.23–41.88	0.028	6.23	1.05–36.93	0.044

*WHO, World Health Organization; OR, odds ratio; 95% CI, 95% confidence interval*.

Finally, we derived eight combinations from the three variate: education, altitude and humidity, and used an altitude <400 m and education >9 years, education >9 years and humidity <70% as the reference combinations. The combination of both altitude >400 m and education <9 years, education <9 years and humidity >70% received the largest effect-size estimate, and all showed statistical significance even after multivariable adjustment.

In view of the magnitude of above interaction, the sample size in this present study was sufficient to derive statistical significance, as revealed by the PS software.

### Prediction Model Construction

**Figure 2:**
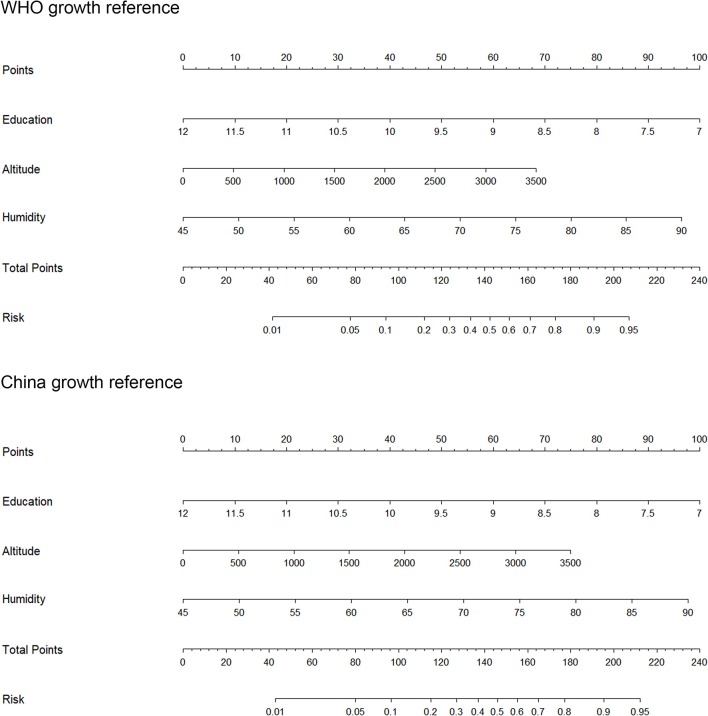
The prediction nomograms of short stature according to the WHO and China references. WHO, World Health Organization.

Based on the significant factors according to the two growth references, we drew a nomogram model to predict the risk of prevalence among short stature (Figure 2). Education, altitude and humidity were included in the nomogram model, which were selected by stepwise, forward and backward logistic regression analyses, according to the two growth references, with *p* < 0.05 (Table 3). For example, based on the WHO growth reference and assuming one place, average education duration was 9 years (60 points), altitude was 500 m (10 points), annual mean relative humidity was 45% (0 points), thus the probability of prevalence of short stature in this area was estimated to be 5% higher than the national prevalence. The predictive accuracy of the two nomogram models was good, and the C-index was 0.832 and 0.835, respectively, according to both WHO and China growth references (all *p* < 0.001).

## Discussion

Our research showed a similar spatial clustering with our previous study in the distribution of short stature by city levels (there were clustering of high prevalence of short stature in the southwest of China and low prevalence in northeast of China) (9). High–low outlier in Suihua City. Low–high outlier in Hanzhong City, Kunming City, the Enshi Autonomous Prefecture and Liuzhou City, Guiyang City and Zhanjiang City. Altitude and humidity increased the risk of high prevalence in short stature, education decreased the risk of high prevalence in short stature. There is interaction effect between altitude and education, humidity and education that lead to the high prevalence of short stature. These three factors can significantly predict the prevalence of short stature in China.

Education is part of the Human Development Index (HDI), which is an index to evaluate human development (34), associated with economic performance and urbanization (35, 36). Numerous studies have reported a closed relationship between education and various diseases such as rheumatoid arthritis, chronic obstructive pulmonary disease, obesity, etc. (37–39). Short stature in children can be attributed to poor nutritional status, repeated infections, and underlying endocrine metabolic and genetic diseases (11, 40, 41). These factors also reflect whether children are well fed and cared for, awareness of the disease and medical care conduct, which are all closely connected to the parents' and societies educational level. Our results in this study are consistent with previously published work that residents' longer education duration may reduce the risk of short stature (19, 22, 23). This represents a good indication for the government that investing in education may effectively reduce the prevalence of short stature.

Our study indicated that children who lived in high-altitude places had a high risk of short stature, which generally applied to other individuals residing in such areas—Peruvians and Tibetans (2, 20), who live in the Andes and Himalayas, respectively, with an average elevation above 4,000 m. Another study focused on the growth retardation of children aged 0–36 months in Tibet showed that with an increase in altitude (3,000 to 4,500 m), the prevalence and odds ratio of stunting gradation increased—this phenomenon was not observed in underweight and wasting (42). This result was also confirmed by studies involving children in Argentina and other countries (43, 44), and may be partly attributed to the negative correlation between birth weight and altitude, where intrauterine growth restrictions result in oxygen deficit and the effect of cytokines, endothelin, etc. (45–49), short stature then continues in postnatal growth (43). Notably, in our study, there are 29 cities where the altitude higher than 1,000 m, it means that plateau hypoxia may not the only reason that lead to the short stature, even though it has been certified that with the altitude rise, SpO_2_ declined (50). Further research into this aspect is also required. In addition, nutrition is another crucial factor that affect the short stature and as it well-known that it has a close relation with the economy. One research focused on the effects of altitude vs. economic status on birth weight and body shape at birth showed that high altitude rather than economic status has close relationship with low birth weight in babies of Bolivia (small for gestational age is one of etiology of short stature) (51).

Another study from Saudi Arabia showed that lowlands (altitude 500 m) also had a high prevalence of short stature (stunting) compared to highlands (altitudes of 2,800–3,150 m), highlighting humidity as another factor affecting short stature (52). Our study also showed that children who lived in places with high relative humidity had a high risk of short stature. It is known that adapting to the high humidity and pathogens in tropical rainforests, consequently results in inhabitants having short stature (1). In part, the underlying mechanism refers to heat stress. Mammals have evolved a heat-regulating center that adjusts to adapt to changes in environmental temperature. When the environmental temperature rises, the body sweats to lose thermal energy, but its efficiency is weakened due to the strong heat stress in a humid environments (18). As a result, short stature in this area can increase the SA:M (surface area:mass) ratio, aiding in heat loss (18). On the other hand, to reduce heat energy in a humid environment, reducing dietary intake accordingly may lead to short body size (18). Scholars have also identified a geno-variation of the GH-IGF-1 pathway in Pygmies who live in tropical rainforest environments and have a short body size, verifying that short stature in rainforest residents may be a selective advantage or adaptive evolution (8, 53).

These data allow us to easily explain the special spatial discrepancy of short stature in Mainland China. First, the high prevalence of short stature in southwest China may be attributed to the education duration of the southwest provinces being lower than those in other parts of China (i.e., 7.76, 7.65, 8.76, and 8.35 years in Yunnan, Guizhou, Guangxi, Sichuan Provinces, respectively), which was 11.71 years in Beijing (17). Second, there was the Yunnan–Guizhou Plateau in southwest China, which covers the provinces of Yunnan and Guizhou (altitude 2,000–4,000 m) (Britannica)^2^. Third, Hainan and Guangxi are coastal provinces that belong to a tropical and subtropical monsoon climate, which is characterized by high temperature and rain. Spatial statistics showing the high–low and low–high outlier areas of the Anselin Local Moran I results were the new findings of this study. Suihua is in the area of the high–low outlier that belongs in Heilongjia Province (46.63°N, 126.98°E). We detected that Suihua City had a high prevalence outlier compared to their neighbors (4.91 and 10.17% according to the WHO and China growth references, respectively). Education years in Suihua City was 8.52 years, which may partly explain this phenomenon, however, further studies are required in this area. On the other hand, low–high outliers like Kunming and Guiyang Cities are provincial capitals, thus the socio-economic pattern of these places may be used as reference for other hotspot areas.

From the above example, we observed that the protective factor of education, and the risk factors of altitude and humidity sometimes overlapped. We therefore estimated the interaction between these factors. Notably, the prevalence in a place where the altitude was >400 m and the education duration was <9 years was approximately twelve times more likely to higher than the national prevalence. The prevalence in a place where the annual relative humidity was >70% and education duration was <9 years was almost six times more likely to higher than the national prevalence. It is easy to see in these interactions that education associated with feeding behaviors and medical care conduct. The prevalence of short stature increased for children living in high risk places (i.e., high altitude or high relative humidity) and residents with short number of education duration. Overall, we developed two predictive nomogram models based on education, altitude and humidity, which can be used to provide accurate predictions for short stature in mainland of China.

We want to convey in this article that some of the children who were diagnosed as short stature in southwest China may show a form of environmental adaption, as in the case of Peruvians and Pygmies. When uniform growth criteria are used for assessing short stature, they may overestimate the prevalence of short stature in children who live in special geographical–climate environments. Beijing attracts many immigrants nationwide, and is the capital of China (40°N, 116°E, located in the north of China). In our pediatric endocrine outpatient department, we often receive children who grew up in southwest China and recently migrated to Beijing. The parents of such children complained that their children were shorter than their classmates who grew in the north of China and wanted them to receive growth hormone treatments. These children were indeed shorter than their classmates, however, if compared to children from southwest China, would this situation change? At present, China has a unique growth reference for children, which was enacted in 2009 (28). We believe that the WHO growth reference faces the same problem when evaluating children from West Africa and Peru. We hope that our medical system will be able to distinguish between normal physiological variation and real disease situation, to avoid overtreatment. Doctors must be prudent when diagnosing short stature for children who come from high risk areas of China (southwest of China). We hope that the new growth reference for China will take the regional difference of height into account. The Yellow Emperor's internal canon, a classic of traditional Chinese medicine said, “when we treat patients, the doctor needs to consider the difference of time, geographical area and individuals in order to give the most suitable strategies for patients,” which is what we wanted to deliver in our study (54).

There are some limitations to our study. First, our study was cross-sectional in nature, and we could not detect and categorize the pathogenetic mechanisms of short stature. In particular, data on the adult height of children, secular increase in the expression of height, the length and weight at birth, the time when the growth rate decreases, the bone age and the size of the parents are not available for us, precluding further analysis or adjustment. Second, our geographical–climate data are interpolation grid data, which may have some different with actual values. Third, our sample size for calculating the interaction effect was small; even though our total sample of students was over 200,000, however, when we generated the prevalence of short stature by cities, the sample size decreased. As a result, the confidence interval became wide. Fourth, we did not collect other potential factors affecting short stature in this study, such as nutritional conditions, repercussions of nutritional alterations, infectious, parasitic, genetic, and velocity of maturation. Fifth, we did not proceed with the gene sequencing for children from southwest China, which may confirm the adaptive evolution of these children. Sixth, as only Chinese school children were analyzed in this present study, extrapolation of our findings to the other racial and ethnic groups is limited.

## Conclusions

Taken together, our findings indicate that spatial clustering was apparent in the distribution of short stature across Chinese cities. High altitude and high humidity are observed to be associated with high prevalence of short stature, and contrastingly, high education was associated with low prevalence. Importantly, altitude, humidity and education can interactively predict the prevalence of short stature in Chinese school children. Further validations are necessary to confirm or refute the conclusions of this present study.

## Data Availability Statement

All datasets generated for this study are included in the manuscript/Supplementary Files.

## Ethics Statement

The written informed consent we have already obtained from parents and children. This study was approved by the Medical Research Ethics Committee of the Peking University Health Science Center (IRB00001052-13082) and the clinical research ethics committee of the China–Japan friendship hospital (2018-94-K68).

## Author Contributions

ZZ, TP, and JuM worked together to develop the study design and analytical plan, revise the manuscript. YD and ZY collated data and calculate the prevalence of short stature from CNSSCH data. WW and CS collected socio-economic and geographical–climate data, conducted spatial statistics. JC and SG analyzed the spatial distribution of short stature. SL conducted the statistical analyses. JiM and WN conducted the statistical analyses and wrote the manuscript. All authors contributed to this work and approved the final manuscript.

### Conflict of Interest

The authors declare that the research was conducted in the absence of any commercial or financial relationships that could be construed as a potential conflict of interest.

## Footnotes

^1^National Science and Technology Infrastructure of China. *National Earth System Science Data Sharing Infrastructure*. Available online at: http://www.geodata.cn

^2^Britannica, T.E.o.E. *Yunnan-Guizhou Plateau*. Available online at: https://www.britannica.com/place/Yunnan-Guizhou-Plateau

## Abbreviations

WHO: World Health Organization
GDP: Gross domestic product
CNSSCH: Chinese National Survey on Students Constitution and Health
HDI: Human Development Index
GH: Growth Hormone
IGF-1: Insulin-like Growth Factor 1.
