# Supplementary material for: Education, Altitude, and Humidity Can Interactively Explain Spatial Discrepancy and Predict Short Stature in 213,795 Chinese School Children
Source: Front Pediatr. 2019 Oct 30;7:425. doi: 10.3389/fped.2019.00425 (PMC6836719; doi:10.3389/fped.2019.00425)
Supplement: Supplementary file 1 [file Data_Sheet_1.pdf]

Tables in the supplementary materials

Supplementary Table 1. The prevalence and standardized prevalence of short stature in cities.

No.	City	WHO growth reference		China growth reference	
		Short stature (%)	Age-Gender standardized rate (%)	Short stature (%)	Age-standardized rate (%)
1	Beijing city	0.33	0.28	0.62	0.64
2	Tianjin city	0.53	0.52	0.75	0.77
3	Shijiazhuang city	1.33	1.30	1.42	1.44
4	Tangshan city	0.63	0.60	0.75	0.73
5	Handan city	1.25	1.18	1.71	1.74
6	Taiyuan city	1.00	0.93	1.63	1.64
7	Datong	2.33	2.36	3.46	3.59
8	Yuncheng city	3.00	2.90	4.17	4.18
9	Hohhot city	0.67	0.67	1.00	1.03
10	Bayannur city	1.45	1.25	1.28	1.23
11	Tongliao city	0.52	0.50	0.69	0.71
12	Chifeng city	2.01	1.77	2.85	2.78
13	Ordos city	1.93	1.72	2.69	2.67
14	Xilingol league	1.41	1.24	2.9	3.06
15	Shenyang city	0.79	0.66	0.71	0.69
16	Dalian city	0.54	0.52	0.83	0.87
17	Fuxin city	1.17	1.00	1.75	1.75
18	Changchun city	1.84	1.71	2.76	2.79
19	Siping city	1.46	1.36	1.58	1.58
20	Baishan city	0.52	0.49	1.08	1.07
21	Harbin city	0.67	0.63	0.83	0.80
22	Qiqihar city	0.76	0.66	0.92	0.92
23	Mudanjiang city	0.42	0.41	0.50	0.51
24	Suihua city	6.02	4.91	10.28	10.17
25	Jiamusi city	2.68	1.81	3.86	3.67
26	Shanghai city	0.60	0.58	0.84	0.82
27	Nanjing city	0.22	0.23	0.57	0.57
28	Wuxi city	0.82	0.81	0.86	0.88

29	Xuzhou city	0.60	0.62	0.73	0.75
30	Hangzhou city	1.25	1.16	1.46	1.45
31	Ningbo city	1.00	0.99	0.88	0.88
32	Jinhua city	1.22	1.21	1.55	1.57
33	Hefei city	1.55	1.53	1.88	1.94
34	Chizhou city	2.54	2.44	3.17	3.15
35	Suzhou city	0.96	0.93	1.25	1.23
36	Fuzhou city	0.46	0.44	0.58	0.58
37	Xiamen city	0.08	0.08	0.08	0.08
38	Nanping city	1.83	1.73	2.71	2.78
39	Jinan city	0.63	0.62	0.67	0.68
40	Yantai city	0.50	0.46	0.42	0.41
41	Jining city	0.17	0.17	0.17	0.18
42	Zhengzhou city	1.50	1.36	1.92	1.94
43	Xinxiang city	1.04	0.95	1.54	1.55
44	Zhoukou city	1.33	1.33	1.83	1.86
45	Changsha city	2.21	2.21	2.47	2.53
46	Hengyang city	5.38	4.94	5.97	5.99
47	Changde city	4.13	3.78	5.96	5.94
48	Guangzhou city	2.25	2.13	2.84	2.83
49	Zhanjiang city	2.88	2.78	3.46	3.42
50	Shaoguan city	6.38	5.97	8.50	8.61
51	Nanning city	3.72	3.52	4.84	4.86
52	Liuzhou city	2.21	2.10	2.38	2.39
53	Wuzhou city	5.21	5.04	5.76	5.69
54	Baise city	6.34	5.82	7.61	7.61
55	Hechi city	11.97	11.40	15.76	15.95
56	Haikou city	5.88	5.18	8.05	7.96
57	County-level administrative units of Hainan province	5.04	4.71	6.38	6.28
58	Chongqing city	5.65	5.37	7.25	7.37
59	Chengdu city	3.21	2.89	4.33	4.36
60	Zigong Hainan province has direct jurisdiction over	7.63	6.86	10.13	10.14
61	Dazhou city	9.04	8.59	11.38	11.49
62	Guiyang city	3.25	3.18	4.00	3.94
63	Tongren city	13.33	12.77	17.83	17.93

64	Bijie city	5.09	4.74	7.76	7.84
65	Qiandongnan miao and dong autonomous prefecture	8.67	8.44	10.75	10.86
66	Qiannan buyi and miao autonomous prefecture	14.67	13.96	20.58	21.07
67	Southwest guizhou buyi miao autonomous prefecture	15.44	13.98	20.12	20.07
68	Kunming city	2.42	2.28	3.34	3.31
69	Dali bai autonomous prefecture	2.92	2.78	4.33	4.27
70	Honghe hani and yi autonomous prefecture	4.96	4.59	6.38	6.35
71	Xi 'an city	2.14	2.13	2.85	2.93
72	Hanzhong city	2.38	2.37	3.17	3.23
73	Yan 'an city	8.84	8.51	12.93	13.20
74	Lanzhou city	1.71	1.65	2.42	2.47
75	Jiuquan city	1.08	1.03	1.71	1.71
76	Tianshui city	4.17	4.23	6.04	6.29
77	Xining city	3.82	3.52	6.20	6.31
78	Yinchuan city	1.54	1.44	2.33	2.35
79	Wuzhong city	1.29	1.14	2.22	2.18
80	Guyuan city	4.02	3.80	5.64	5.76
81	Urumqi city	1.00	0.95	1.08	1.07
82	Yili kazakh autonomous prefecture	1.08	1.07	1.67	1.67
83	Aksu Region	1.92	1.88	2.67	2.67
84	Kashgar Region	5.82	5.05	8.73	8.79
85	Kzil sukotz autonomous prefecture	1.67	1.67	2.90	2.95
86	Altay Region	5.08	4.43	7.67	7.48
87	Wuhan city	1.92	1.97	2.90	2.94
88	Huangshi city	0.82	0.86	1.12	1.14
89	Xiangyang city	0.82	0.86	1.12	1.14
90	Xiaogan city	0.82	0.86	1.12	1.14
91	Enshi autonomous prefecture	0.82	0.86	1.12	1.14
92	Jingzhou city	2.35	2.39	3.53	3.67

93	Shiyan city	2.35	2.39	3.53	3.67
94	Xianning city	2.35	2.39	3.53	3.67
95	Xiantao city	2.35	2.39	3.53	3.67
96	Nanchang city	2.84	2.82	3.67	3.75
97	Jingdezhen city	2.84	2.82	3.67	3.75
98	Shangrao city	2.84	2.82	3.67	3.75
99	Fuzhou city	2.84	2.82	3.67	3.75
100	Ganzhou city	2.43	2.40	3.02	3.01
101	Xinyu city	2.43	2.40	3.02	3.01
102	Ji'an city	2.43	2.40	3.02	3.01
103	Yingtan city	2.43	2.40	3.02	3.01
104	Jiujiang city	3.67	3.67	5.29	5.42
105	Pingxiang city	3.67	3.67	5.29	5.42
106	Yichun city	3.67	3.67	5.29	5.42

Abbreviations: WHO, World Health Organization.

Supplementary Table 2. The results of Getis-Ord Gi*.

City	WHO growth reference			China growth reference		
	Z Score	P Value	Gi_Bin	Z Score	P Value	Gi_Bin
Qiqihar city	-1.33	0.183	0	-0.97	0.332	0
Jiamusi city	-0.97	0.334	0	-0.51	0.607	0
Altay Region	-0.02	0.982	0	0.11	0.909	0
Mudanjiang city	-1.33	0.183	0	-0.94	0.349	0
Xiantao city	-0.52	0.600	0	-0.31	0.758	0
Changchun city	-1.90	0.058	-1	-1.54	0.123	0
Urumqi city	-0.50	0.620	0	-0.37	0.711	0
Xilingol league	-2.00	0.046	-2	-1.80	0.072	-1
Tongliao city	-2.31	0.021	-2	-1.93	0.054	-1
Yili kazakh autonomous prefecture	-0.87	0.385	0	-0.86	0.388	0
Siping city	-2.04	0.041	-2	-1.76	0.079	-1
Chifeng city	-2.28	0.023	-2	-2.12	0.034	-2
Baishan city	-1.84	0.065	-1	-1.55	0.120	0
Shenyang city	-2.40	0.016	-2	-2.13	0.033	-2
Fuxin city	-2.65	0.008	-3	-2.50	0.013	-2
Aksu Region	-0.52	0.600	0	-0.31	0.758	0
Bayannur city	-0.52	0.603	0	-0.35	0.729	0
Hohhot city	-1.59	0.111	0	-1.38	0.169	0
Kzil sukotz autonomous prefecture	0.01	0.994	0	0.29	0.773	0
Beijing city	-3.17	0.002	-3	-3.05	0.002	-3
Datong	-1.98	0.048	-2	-1.83	0.068	-1
Ordos city	-0.59	0.552	0	-0.46	0.642	0
Tangshan city	-3.07	0.002	-3	-2.96	0.003	-3
Yinchuan city	-0.22	0.829	0	0.01	0.995	0
Kashgar Region	0.01	0.994	0	0.29	0.773	0
Taiyuan city	-1.99	0.047	-2	-1.88	0.060	-1
Wuzhong city	-0.22	0.829	0	0.01	0.995	0
Shijiazhuang city	-2.28	0.023	-2	-2.22	0.026	-2
Yantai city	-2.89	0.004	-3	-2.94	0.003	-3
Jinan city	-3.07	0.002	-3	-3.08	0.002	-3
Xining city	-0.34	0.737	0	-0.06	0.952	0
Handan city	-2.46	0.014	-2	-2.43	0.015	-2
Guyuan city	0.73	0.462	0	0.94	0.350	0
Xinxiang city	-2.29	0.022	-2	-2.20	0.028	-2
Tianshui city	1.12	0.264	0	1.28	0.200	0

Zhengzhou city	-2.08	0.038	-2	-1.99	0.046	-2
Xuzhou city	-3.00	0.003	-3	-3.11	0.002	-3
Xi 'an city	-0.23	0.821	0	-0.09	0.928	0
Suzhou city	-2.85	0.004	-3	-2.98	0.003	-3
Hanzhong city	2.12	0.034	2	2.18	0.030	2
Zhoukou city	-1.93	0.053	-1	-1.90	0.057	-1
Shiyan city	0.94	0.349	0	0.93	0.355	0
Xiangyang city	0.36	0.723	0	0.17	0.865	0
Dazhou city	3.76	0.000	3	3.76	0.000	3
Nanjing city	-2.45	0.014	-2	-2.62	0.009	-3
Wuxi city	-2.22	0.026	-2	-2.40	0.016	-2
Wuhan city	-1.95	0.051	-1	-2.16	0.031	-2
Xiaogan city	-1.23	0.221	0	-1.48	0.138	0
Chengdu city	2.63	0.009	3	2.50	0.012	2
Chizhou city	-2.22	0.026	-2	-2.43	0.015	-2
Jingzhou city	1.30	0.194	0	0.90	0.368	0
Huangshi city	-1.58	0.113	0	-1.81	0.071	-1
Enshi autonomous prefecture	2.83	0.005	3	2.63	0.009	3
Chongqing city	4.82	0.000	3	4.56	0.000	3
Hangzhou city	-2.08	0.037	-2	-2.29	0.022	-2
Jiujiang city	-1.80	0.072	-1	-2.05	0.041	-2
Zigong Hainan province has direct jurisdiction over	6.06	0.000	3	5.85	0.000	3
Ningbo city	-1.52	0.129	0	-1.72	0.086	-1
Xianning city	-0.51	0.608	0	-0.76	0.445	0
Jingdezhen city	-2.05	0.041	-2	-2.31	0.021	-2
Changde city	2.27	0.023	2	1.85	0.065	1
Shangrao city	-1.42	0.156	0	-1.68	0.093	-1
Jinhua city	-1.91	0.056	-1	-2.14	0.032	-2
Nanchang city	-1.64	0.101	0	-1.91	0.056	-1
Changsha city	1.11	0.266	0	0.66	0.510	0
Tongren city	5.32	0.000	3	4.94	0.000	3
Fuzhou city	-1.17	0.242	0	-1.45	0.148	0
Nanping city	-1.37	0.171	0	-1.67	0.094	-1
Yingtan city	-1.29	0.197	0	-1.55	0.121	0
Yichun city	-1.19	0.233	0	-1.42	0.155	0
Xinyu city	-1.19	0.233	0	-1.42	0.155	0
Bijie city	6.14	0.000	3	5.82	0.000	3
Pingxiang city	0.44	0.660	0	-0.01	0.990	0
Qiandongnan miao and dong	6.14	0.000	3	5.70	0.000	3

autonomous prefecture						
Ganzhou city	-0.21	0.831	0	-0.57	0.572	0
Hengyang city	2.61	0.009	3	2.15	0.032	2
Guiyang city	6.39	0.000	3	5.92	0.000	3
Ji'an city	-0.48	0.630	0	-0.84	0.401	0
Dali bai autonomous prefecture	2.90	0.004	3	2.98	0.003	3
Qiannan buyi and miao autonomous prefecture	6.50	0.000	3	6.02	0.000	3
Liuzhou city	5.48	0.000	3	4.99	0.000	3
Fuzhou city	-0.72	0.470	0	-0.97	0.331	0
Kunming city	5.39	0.000	3	5.25	0.000	3
Southwest guizhou buyi miao autonomous prefecture	6.25	0.000	3	5.88	0.000	3
Xiamen city	-0.23	0.820	0	-0.51	0.612	0
Hechi city	6.38	0.000	3	5.92	0.000	3
Baise city	5.91	0.000	3	5.46	0.000	3
Honghe hani and yi autonomous prefecture	4.73	0.000	3	4.65	0.000	3
Wuzhou city	5.61	0.000	3	5.00	0.000	3
Guangzhou city	1.57	0.117	0	1.11	0.268	0
Zhanjiang city	2.59	0.010	3	2.17	0.030	2
Haikou city	2.01	0.044	2	1.64	0.102	0
Suihua city	-1.73	0.084	-1	-1.39	0.165	0
Harbin city	-1.73	0.084	-1	-1.39	0.165	0
Tianjin city	-3.16	0.002	-3	-3.04	0.002	-3
Dalian city	-2.70	0.007	-3	-2.68	0.007	-3
Yan 'an city	-0.64	0.519	0	-0.57	0.565	0
Lanzhou city	0.38	0.703	0	0.65	0.514	0
Jining city	-3.25	0.001	-3	-3.30	0.001	-3
Yuncheng city	-1.33	0.185	0	-1.24	0.214	0
Hefei city	-2.59	0.009	-3	-2.78	0.005	-3
Shanghai city	-1.83	0.067	-1	-2.03	0.042	-2
Shaoguan city	1.25	0.211	0	0.77	0.444	0
Nanning city	6.24	0.000	3	5.74	0.000	3
Jiuquan city	-0.24	0.809	0	0.02	0.984	0
County-level administrative units of Hainan province	1.26	0.206	0	0.97	0.332	0
Xiantao city	1.69	0.090	1	1.51	0.130	0

Abbreviations: WHO, World Health Organization.

Supplementary Table 3. The results of Anselin Local Moran I.

City	WHO growth reference			China growth reference		
	Z Score	P Value	Type	Z Score	P Value	Type
Qiqihar city	0.51	0.332	Not Significant	-0.10	0.398	Not Significant
Jiamusi city	0.92	0.168	Not Significant	0.53	0.364	Not Significant
Altay Region	-0.66	0.236	Not Significant	-0.75	0.176	Not Significant
Mudanjiang city	1.20	0.1	Not Significant	0.76	0.252	Not Significant
Xiantao city	0.37	0.456	Not Significant	0.35	0.42	Not Significant
Changchun city	1.70	0.02	LL	1.38	0.042	LL
Urumqi city	0.10	0.434	Not Significant	-0.07	0.378	Not Significant
Xilingol league	1.80	0.004	LL	1.68	0.012	LL
Tongliao city	1.97	0.006	LL	1.73	0.016	LL
Yili kazakh autonomous prefecture	0.56	0.322	Not Significant	0.59	0.322	Not Significant
Siping city	1.87	0.008	LL	1.53	0.028	LL
Chifeng city	2.18	0.002	LL	2.03	0.002	LL
Baishan city	1.80	0.01	LL	1.60	0.02	LL
Shenyang city	1.91	0.002	LL	1.77	0.004	LL
Fuxin city	2.15	0.002	LL	2.11	0.002	LL
Aksu Region	0.17	0.454	Not Significant	0.13	0.414	Not Significant
Bayannur city	0.59	0.304	Not Significant	0.49	0.36	Not Significant
Hohhot city	1.39	0.052	Not Significant	1.19	0.122	Not Significant
Kzil sukotz autonomous prefecture	-0.58	0.206	Not Significant	-0.83	0.162	Not Significant
Beijing city	2.61	0.002	LL	2.39	0.002	LL
Datong	2.16	0.002	LL	2.08	0.006	LL
Ordos city	0.64	0.278	Not Significant	0.57	0.338	Not Significant
Tangshan city	2.58	0.002	LL	2.49	0.002	LL
Yinchuan city	0.36	0.406	Not Significant	0.05	0.436	Not Significant
Kashgar Region	-0.52	0.354	Not Significant	-0.38	0.436	Not Significant
Taiyuan city	1.64	0.034	LL	1.46	0.042	LL
Wuzhong city	0.04	0.424	Not Significant	-0.20	0.364	Not Significant
Shijiazhuang city	2.25	0.002	LL	2.14	0.004	LL
Yantai city	2.62	0.002	LL	2.92	0.002	LL
Jinan city	2.92	0.002	LL	3.04	0.002	LL
Xining city	-0.45	0.388	Not Significant	-0.24	0.474	Not Significant
Handan city	2.45	0.002	LL	2.39	0.002	LL
Guyuan city	0.46	0.286	Not Significant	0.74	0.218	Not Significant
Xinxiang city	1.96	0.006	LL	1.89	0.014	LL

Tianshui city	0.90	0.178	Not Significant	0.93	0.172	Not Significant
Zhengzhou city	1.85	0.012	LL	1.75	0.024	LL
Xuzhou city	2.18	0.002	LL	2.40	0.002	LL
Xi 'an city	-0.31	0.334	Not Significant	-0.56	0.272	Not Significant
Suzhou city	2.45	0.002	LL	2.45	0.002	LL
Hanzhong city	-2.14	0.032	LH	-2.15	0.03	LH
Zhoukou city	2.17	0.008	LL	2.15	0.008	LL
Shiyan city	-0.28	0.342	Not Significant	-0.32	0.336	Not Significant
Xiangyang city	0.17	0.442	Not Significant	0.13	0.478	Not Significant
Dazhou city	2.61	0.014	HH	2.51	0.016	HH
Nanjing city	2.37	0.004	LL	2.38	0.002	LL
Wuxi city	2.15	0.004	LL	2.25	0.002	LL
Wuhan city	1.47	0.04	LL	1.51	0.05	LL
Xiaogan city	1.02	0.164	Not Significant	1.13	0.11	Not Significant
Chengdu city	3.10	0.008	HH	2.78	0.01	HH
Chizhou city	1.83	0.018	LL	1.98	0.016	LL
Jingzhou city	-0.28	0.35	Not Significant	-0.15	0.4	Not Significant
Huangshi city	0.75	0.242	Not Significant	0.91	0.17	Not Significant
Enshi autonomous prefecture	-3.17	0.006	LH	-2.79	0.014	LH
Chongqing city	4.52	0.002	HH	4.36	0.002	HH
Hangzhou city	1.95	0.014	LL	2.05	0.006	LL
Jiujiang city	-1.22	0.116	Not Significant	-1.30	0.078	Not Significant
Zigong Hainan province has direct jurisdiction over	4.48	0.002	HH	4.58	0.002	HH
Ningbo city	1.60	0.032	LL	1.77	0.014	LL
Xianning city	0.60	0.302	Not Significant	0.77	0.234	Not Significant
Jingdezhen city	-1.21	0.106	Not Significant	1.35	0.068	Not Significant
Changde city	1.33	0.106	Not Significant	1.01	0.164	Not Significant
Shangrao city	-0.81	0.212	Not Significant	1.04	0.142	Not Significant
Jinhua city	1.70	0.016	LL	1.93	0.004	LL
Nanchang city	-0.70	0.264	NotSignificant	0.96	0.166	Not Significant
Changsha city	-0.87	0.206	Not Significant	-0.62	0.262	Not Significant
Tongren city	5.52	0.002	HH	5.16	0.002	HH
Fuzhou city	-0.78	0.214	Not Significant	0.93	0.18	Not Significant
Nanping city	1.16	0.112	Not Significant	1.48	0.052	Not Significant
Yingtan city	0.76	0.228	Not Significant	0.89	0.178	Not Significant
Yichun city	-0.49	0.346	Not Significant	-0.79	0.23	Not Significant
Xinyu city	0.04	0.488	Not Significant	0.33	0.412	Not Significant
Bijie city	6.30	0.002	HH	6.11	0.002	HH
Pingxiang city	0.38	0.336	Not Significant	-0.20	0.456	Not Significant

Qiandongnan miao and dong autonomous prefecture	6.83	0.002	HH	6.54	0.002	HH
Ganzhou city	-0.14	0.408	Not Significant	0.22	0.466	Not Significant
Hengyang city	2.22	0.02	HH	1.69	0.06	Not Significant
Guiyang city	7.55	0.002	HH	-7.04	0.002	LH
Ji'an city	-0.04	0.446	Not Significant	0.28	0.418	Not Significant
Dali bai autonomous prefecture	2.55	0.024	HH	2.38	0.034	HH
Qiannan buyi and miao autonomous prefecture	6.48	0.002	HH	5.45	0.002	HH
Liuzhou city	-6.56	0.002	LH	-5.77	0.002	LH
Fuzhou city	-0.78	0.214	Not Significant	1.03	0.154	Not Significant
Kunming city	-4.93	0.002	LH	-5.27	0.002	LH
Southwest guizhou buyi miao autonomous prefecture	5.82	0.002	HH	5.43	0.002	HH
Xiamen city	0.40	0.378	Not Significant	0.63	0.28	Not Significant
Hechi city	6.19	0.002	HH	5.60	0.002	HH
Baise city	6.03	0.002	HH	5.98	0.002	HH
Honghe hani and yi autonomous prefecture	4.13	0.002	HH	4.13	0.002	HH
Wuzhou city	4.42	0.004	HH	3.93	0.002	HH
Guangzhou city	-1.67	0.07	Not Significant	-1.12	0.138	Not Significant
Zhanjiang city	2.32	0.034	HH	-2.01	0.046	LH
Haikou city	1.24	0.108	Not Significant	0.90	0.148	Not Significant
Suihua city	-1.84	0.004	HL	-1.86	0.002	HL
Harbin city	1.16	0.106	Not Significant	0.57	0.33	Not Significant
Tianjin city	2.56	0.002	LL	2.51	0.002	LL
Dalian city	2.43	0.002	LL	2.58	0.002	LL
Yan 'an city	-0.97	0.18	Not Significant	-0.92	0.17	Not Significant
Lanzhou city	-0.41	0.31	Not Significant	-0.70	0.202	Not Significant
Jining city	3.12	0.002	LL	2.86	0.002	LL
Yuncheng city	-0.85	0.204	Not Significant	-0.73	0.252	Not Significant
Hefei city	2.43	0.002	LL	2.78	0.002	LL
Shanghai city	1.78	0.012	LL	2.00	0.004	LL
Shaoguan city	0.73	0.22	Not Significant	0.26	0.372	Not Significant
Nanning city	5.90	0.002	HH	5.50	0.002	HH
Jiuquan city	-0.32	0.238	Not Significant	-0.57	0.16	Not Significant
County-level administrative units of Hainan province	0.93	0.136	Not Significant	0.94	0.162	Not Significant
Xiantao city	0.37	0.456	Not Significant	0.60	0.308	Not Significant

Abbreviations: WHO, World Health Organization; HH：High-High cluster; LL: Low-Low cluster; HL: High-Low cluster; LH:Low-High cluste.
